# Non-ST Elevation Myocardial Infarction

**DOI:** 10.31083/RCM52864

**Published:** 2026-10-13

**Authors:** Miro Asadourian, John A. Ambrose

**Affiliations:** ^1^Department of Cardiovascular Medicine, UCSF-Fresno, Fresno, CA 93701-2302, USA

**Keywords:** NSTEMI, Type 1 MI, Type 2 MI, revascularization

## Abstract

Non-ST-elevation myocardial infarction (NSTEMI) is the most common acute coronary syndrome presentation and represents a heterogeneous clinical entity with distinct mechanisms and distinct diagnostic and therapeutic implications. This narrative review summarizes contemporary concepts in NSTEMI pathophysiology, classification, angiographic phenotypes, and treatment strategies, with an emphasis on differentiating Type 1 from Type 2 myocardial infarction (MI) and the implications for revascularization decisions. The PubMed/MEDLINE database was searched for English-language publications addressing NSTEMI pathophysiology, classification, diagnosis, and management, supplemented by the most recent American and European acute coronary syndrome guidelines, the Universal Definition of Myocardial Infarction, landmark randomized controlled trials, and pivotal mechanistic and intravascular imaging studies, with priority given to contemporary and high-impact sources. NSTEMI emerges as a heterogeneous syndrome rather than a single disease, and optimal care depends on identifying the underlying mechanism. This requires distinguishing myocardial infarction from myocardial injury, separating Type 1 atherothrombosis from Type 2 supply–demand mismatch, and recognizing entities such as myocardial infarction with non-obstructive coronary arteries and spontaneous coronary artery dissection. A mechanism-specific approach should guide antithrombotic therapy and revascularization decisions, and the transition from a syndromic label to an individualized, mechanism-based diagnosis is essential for optimizing patient outcomes.

## 1. Introduction

Non-ST-elevation myocardial infarction (NSTEMI) is now the most common presentation of acute coronary syndrome, accounting for most cases of myocardial infarction in large registries [1,2,3]. Defined electrocardiographically by the absence of persistent ST-segment elevation, NSTEMI is not a single disease entity but a final common phenotype shared by patients with markedly different underlying mechanisms. While several specific subtypes have been identified, challenges remain in nomenclature, diagnosis, and treatment. In this review, we summarize the pathophysiology, classification, epidemiology, and angiographic phenotypes of NSTEMI and discuss mechanism-specific treatment.

## 2. Literature Search

This narrative review is based on a search of the English-language literature indexed in PubMed/MEDLINE from database inception through 2026, supplemented by manual review of the reference lists from key articles and current society guideline documents. Search terms included combinations of “NSTEMI”, “non-ST-elevation acute coronary syndrome”, “Type 1 myocardial infarction”, “Type 2 myocardial infarction”, “myocardial injury”, “universal definition”, “MINOCA”, “spontaneous coronary artery dissection”, “revascularization”, “dual antiplatelet therapy”, and “intravascular imaging”. Priority was given to randomized controlled trials, large registries, contemporary society guidelines, and authoritative review articles and scientific statements. Given the narrative format, the selection of cited literature reflects the judgment of relevance by the authors rather than adherence to a formal systematic review protocol.

## 3. NSTEMI: Pathophysiology and Treatment

### 3.1 Historical Perspective

For much of the 20th century, the immediate cause of acute myocardial infarction (AMI) was debated in the literature. A thrombus was identified at autopsy in most, but not all, cases, raising the question of whether it was the primary cause or secondary to reduced coronary blood flow from another mechanism. This debate was effectively settled in 1973 at a National Institutes of Health workshop attended by leading pathologists, clinicians, cardiologists, and hematologists. The consensus conclusion was that the weight of evidence supported a primary role for intracoronary thrombus as the immediate cause of transmural myocardial infarction (MI) at autopsy, a finding that closely correlates with the current definition of ST-elevation MI (STEMI) [4].

However, pathologic analyses at autopsy demonstrated that not every patient dying from AMI had either transmural necrosis or intracoronary thrombus. In many such cases, necrosis was partial and predominantly involved the inner layers of the myocardium. These were classified as non-transmural or subendocardial infarctions [5]. In the 1970s and 1980s, clinical and electrocardiogram (ECG) criteria were developed for non-transmural MI and included chest pain typical of myocardial ischemia lasting >15 minutes, new persistent ST depression or T-wave inversions, and elevated cardiac enzyme levels without new Q waves [6]. This definition was criticized as misleading because it did not consistently correlate with pathologic findings. Patients classified as having non-transmural infarction by ECG could have transmural infarction at autopsy. In the 1990s, in part to shift from a pathologic to a clinical classification, acute MI was redefined as ST-elevation versus non-ST-elevation MI (STEMI vs. NSTEMI), and the earlier terms were largely discarded. This designation persists today, and various types of NSTEMI have been recognized based on differing mechanisms. These mechanisms are discussed in the next section.

The 2007 American College of Cardiology (ACC)/American Heart Association (AHA) guidelines for the management of patients with non-ST-elevation acute coronary syndrome (ACS) provide excellent definitions of NSTEMI pathophysiology, summarized as follows [7].

(1) The most common underlying molecular and cellular pathophysiology of disrupted atherosclerotic plaque (the predominant mechanism of STEMI and NSTEMI) is arterial inﬂammation, typically triggered by noninfectious stimuli (*e*.*g*., oxidized lipids), which can lead to plaque expansion and destabilization, rupture or plaque erosion, and thrombogenesis. Activated macrophages and T lymphocytes at the shoulder of the plaque increase the expression of enzymes, such as metalloproteinases, that cause thinning and disruption of the plaque, potentially leading to plaque rupture and subsequent unstable angina (UA)/NSTEMI (as well as STEMI).

(2) A less common mechanism is dynamic coronary obstruction, triggered by intense focal spasm of a segment of an epicardial coronary artery (Prinzmetal’s angina). This local spasm results from hypercontractility of vascular smooth muscle and/or endothelial dysfunction. Large-vessel spasm may occur on top of obstructive or destabilized plaque. Dynamic coronary obstruction can also arise from diffuse microvascular dysfunction; for example, due to endothelial dysfunction or abnormal constriction of small intramural resistance vessels. Coronary spasm is also the presumed mechanism underlying cocaine-induced UA/NSTEMI.

(3) Another mechanism is severe fixed coronary narrowing with or without superimposed spasm or thrombus. This occurs in some patients with progressive atherosclerosis or with restenosis after percutaneous coronary intervention (PCI).

(4) Spontaneous coronary artery dissection (SCAD) is an important cause of NSTEMI, particularly in women.

(5) A further common cause of NSTEMI is myocardial necrosis due to a supply–demand mismatch extrinsic to the coronary vascular bed. Patients may or may not have underlying coronary artery disease, and the NSTEMI is precipitated by conditions such as acute anemia, hypotension, severe hypoxia, tachycardia, heart failure, or severe hypertension.

(6) Additional categories not included in the 2007 summary include myocardial infarction with non-obstructive coronary arteries (MINOCA) [8]. This category may overlap with several of the mechanisms above but will be discussed separately later in this review. Furthermore, the pathophysiology of MI has been expanded to include plaque erosion and calcified nodules. A defect in the endothelium with overlying thrombus (plaque erosion) has been increasingly recognized on intravascular imaging, particularly optical coherence tomography (OCT), in NSTEMI [9]. Small OCT studies in selected patients with NSTEMI suggest that plaque erosion may be a common cause of NSTEMI, occurring in approximately 40% of cases, which is higher than the proportion observed in STEMI [9]. Calcified nodules as a cause of MI (either STEMI or NSTEMI) are more often seen in older patients or those with renal failure, in whom intracoronary thrombus formation develops on nodular calcium protruding into the vessel lumen [10].

### 3.2 NSTEMI Epidemiology

According to a 2025 review in The Cardiology Advisor (February 13, 2025), there are roughly 780,000 cases of ACS annually in the United States (US), and approximately 70% (≈546,000) are NSTEMI. Males outnumber females by about 3:2, consistent with broader ACS epidemiology [1]. These estimates are based on population-level data using contemporary definitions of MI and encompass all etiologic subtypes classified under the Universal Definition (see below). Data from National Cardiovascular Data Registry (NCDR) programs provide two complementary “real-world” views. First, in the CathPCI Registry (patients undergoing PCI), 667,424 PCI procedures were performed in 2014 across 1612 US hospitals. Among indications, NSTEMI was more common than STEMI (≈17.9% vs. 15.8% of all PCIs or about 118,000 NSTEMI PCIs), approximating the annual volume of Type 1 NSTEMI cases that proceed to revascularization in this registry [2]. Second, in the later Chest Pain–MI Registry (all MI presentations at participating centers) between January 2019 and June 2020, 325,783 MI cases were recorded, including 91,262 STEMIs—implying that the majority (234,521) were NSTEMIs [3]. While these numbers do not capture all NSTEMI cases in the US (because of non-participating hospitals), these data underscore that NSTEMI is substantially more frequent than STEMI. Moreover, these data do not include the vast majority of Type 2 NSTEMIs (see below).

Risk factors for coronary artery disease are highly prevalent in NSTEMI cohorts and include hypertension, hyperlipidemia, diabetes, smoking, obesity, and family history. For example, registry data show diabetes in 36–41% of NSTEMI patients, atrial fibrillation in ~9–12%, and a declining prevalence of active smoking over the last decade [11]. The mean or median age at presentation depends on the overall risk profile but generally falls in the mid-60s to early-70s, with a US median age of approximately 68 years for ACS overall [11].

### 3.3 Universal Definitions of Myocardial Infarction

With the advent of increasingly sensitive troponin assays, the specificity of elevated troponin for myocardial infarction declined. Clinicians were frequently consulted about hospitalized patients with troponin levels above the upper reference limit but without signs or symptoms of cardiac ischemia. The key questions became: What is the correct diagnosis, and are all such patients considered to have an MI?

In 2007, the First Universal Definition of Myocardial Infarction (UDMI) was published to clarify the diagnosis of MI in the context of elevated troponin levels [12]. The UDMI divided myocardial infarction into five categories (see Table 1). A diagnosis of MI required a rise and/or fall of troponin values with at least one value above the 99th-percentile upper reference limit, together with clinical evidence of myocardial ischemia based on one or more of the following: ischemic symptoms, new ischemic ECG changes, development of pathological Q waves, or imaging evidence of new loss of viable myocardium or new regional wall-motion abnormality. In the absence of such clinical evidence, an elevated troponin level should be attributed to myocardial injury rather than infarction. The Fourth UDMI, published in 2018, did not substantially change this overarching framework [13].

**Table 1.** **Summary of the Universal Definition framework as applied to NSTEMI, organizing MI into its major types (Type 1–5) based on the underlying mechanism**.

Type	Universal definition (core criteria)	Typical mechanism/clinical context
NSTEMI *Umbrella clinical diagnosis*	Acute MI: rise/fall in cardiac troponin, with at least one value >99th percentile URL plus evidence of acute myocardial ischemia (ischemic symptoms, new ischemic ECG changes, new Q waves, new imaging abnormality, or coronary thrombus), but without STEMI ECG criteria.	Non-ST-elevation ACS; mechanism depends on MI type (Type 1 vs. Type 2 vs. procedure-related).
Type 1 MI *Spontaneous*	MI due to a primary coronary event (*e*.*g*., plaque rupture/fissuring/erosion) resulting in intraluminal thrombus.	Atherothrombotic ACS; often with a culprit lesion; may present as NSTEMI.
Type 2 MI *Supply–demand mismatch*	MI secondary to an imbalance between myocardial oxygen supply–demand (increased demand and/or decreased supply) not related to acute coronary atherothrombosis. Includes epicardial causes such as SCAD, epicardial coronary spasm, and coronary embolism.	Ischemia from physiological or hemodynamic triggers (tachyarrhythmia, sepsis, hypoxemia, anemia/bleeding, hypotension/shock, severe hypertension, spasm/embolism); CAD may coexist. No acute thrombotic culprit lesion from plaque rupture/erosion is present.
Type 3 MI *Sudden cardiac death*	Cardiac death with symptoms suggestive of myocardial ischemia and presumed new ischemic ECG changes or ventricular fibrillation, but death occurs before biomarkers can be obtained or before troponin rises.	Fatal ischemic event prior to biomarker confirmation.
Type 4a MI *PCI-related*	MI temporally related to PCI (meeting Universal Definition troponin thresholds) with supporting evidence of acute myocardial ischemia beyond isolated injury.	Periprocedural ischemia/injury (*e*.*g*., side-branch occlusion, no-reflow, dissection, thrombosis).
Type 4b MI *Stent/scaffold thrombosis*	MI associated with stent/scaffold thrombosis documented by angiography or autopsy.	Acute device thrombosis; may present as NSTEMI or STEMI.
Type 4c MI *Restenosis*	MI associated with restenosis after PCI.	Flow-limiting restenosis (often in-stent restenosis) leading to ischemia; may present as NSTEMI.
Type 5 MI *CABG-related*	MI temporally related to CABG (meeting Universal Definition troponin thresholds) with supporting evidence of acute myocardial ischemia.	Perioperative ischemia/injury (*e*.*g*., graft failure, embolization, hypotension).

NSTEMI, non-ST-elevation myocardial infarction; MI, myocardial infarction; STEMI, ST-elevation MI; SCAD, spontaneous coronary artery dissection; URL, upper reference limit (in this context, the 99th percentile upper reference limit for cardiac troponin); ECG, electrocardiogram; ACS, acute coronary syndrome; CAD, coronary artery disease; PCI, percutaneous coronary intervention; CABG, coronary artery bypass grafting.

This article mainly focuses on the two most important categories for clinical practice, Type 1 and Type 2 MI, which represent the most common presentations of MI in hospitalized patients. NSTEMI occurring after PCI or coronary artery bypass grafting (CABG) is not addressed in this article.

**Type 1 MI**. Type 1 MI corresponds to the first category described in the prior 2007 ACC/AHA guidelines. According to the UDMI, Type 1 is caused by atherothrombosis of an epicardial artery due to plaque rupture, plaque erosion, or a calcified nodule [12]. Thrombotic total occlusion of an epicardial coronary artery from these pathophysiological mechanisms is most common in STEMI, whereas total occlusion is less common in Type 1 NSTEMI.

Intravascular imaging, particularly with OCT performed during invasive coronary angiography immediately before percutaneous coronary intervention, has been crucial for understanding the *in vivo* pathophysiology of Type 1 MI. With an axial resolution of approximately 10–15 μm, OCT can reliably distinguish most cases of plaque rupture from plaque erosion or calcified nodules. Type 1 NSTEMIs share the same fundamental mechanisms as STEMIs, but as noted above, the culprit artery is less likely to be totally occluded. When total occlusion is present, it may involve a smaller myocardial territory (*e*.*g*., a branch vessel), a vessel with established collateral circulation, or a vessel with intermittent total occlusion. In other cases, multivessel coronary disease may be present, and a single culprit lesion or artery may be difficult or impossible to identify.

**Type 2 MI**. According to the UDMI, Type 2 MI refers to an MI (either STEMI or NSTEMI) in which myocardial ischemia/necrosis with troponin elevation is primarily caused by factors extrinsic to the coronary vascular bed, such as acute severe anemia, hypotension, severe hypoxia, leading to a significant supply–demand mismatch. The UDMI also includes epicardial causes that are not due to acute coronary atherothrombosis, including spontaneous coronary artery dissection (SCAD), coronary spasm, and coronary embolization, as potential mechanisms for Type 2 MI. Differentiation between Type 1 and Type 2 MI is necessary because management strategies differ between these entities. However, in many cases, the distinction between Types 1 and 2 is not apparent, leading to diagnostic uncertainty and challenges in therapeutic decision-making.

#### Problems With Type 2 Diagnosis

An elevation in troponin above the upper reference limit may reflect either myocardial injury or myocardial infarction. When this biomarker is applied to the diagnosis of Type 2 MI, there is marked non-uniformity. Across the literature, the reported incidence of Type 2 MI among patients with acute MI ranges from <5% to >70% [14]. Thus, it is unlikely that such a wide range can be explained solely by baseline differences in the studied population. We believe several problems inherent to the current Type 2 MI definition contribute to this variability. Furthermore, a recent systematic review has highlighted that Type 2 MI remains underrepresented or inadequately distinguished from Type 1 MI in major NSTEMI clinical trials, underscoring the need for more precise enrollment criteria and dedicated therapeutic studies for this population [15].

Based on expert opinion, we previously summarized the difficulties with a Type 2 diagnosis in an article published in the American Journal of Cardiology [16]. These include the following:

(1) According to the UDMI, neither the degree of supply–demand mismatch nor the type of symptoms is standardized for Type 2 MI diagnosis.

(2) Troponin values that fluctuate just above and below the upper reference limit can be interpreted as a flat pattern rather than a clear rise and fall. All of these issues promote subjective interpretation and, in turn, diagnostic non-uniformity.

(3) Symptoms may vary considerably, further confusing clinicians. Studies reporting a very high incidence of Type 2 MI may classify heart failure patients who present with a small troponin increase and dyspnea as the only clinical evidence of myocardial ischemia as having myocardial infarction, rather than diagnosing these patients with myocardial injury [17]. While dyspnea can be an anginal equivalent, the near-universal presence of this condition in heart failure exacerbations renders this complaint highly nonspecific. Thus, Type 2 MI can be both overdiagnosed and underdiagnosed. In one study, patients with unequivocal Type 2 MI, as determined by experienced cardiologists on retrospective chart review, were compared with hospital discharge diagnoses in a blinded fashion; fewer than 10% were correctly diagnosed during the index hospitalization [17].

(4) Additional issues with the UDMI definition of Type 2 MI relate to the inclusion of epicardial conditions such as SCAD, epicardial spasm, and coronary embolization. Prior studies have identified factors extrinsic to the coronary vascular bed as causes of acute coronary syndromes. In 1939, a pathological study described patients with myocardial necrosis in the absence of intracoronary thrombus, suggesting that the necrosis might have been caused by factors extrinsic to the coronary vascular bed [5]. Similarly, in 1989, Braunwald [18], in his classic paper on unstable angina, classified some cases as secondary to extrinsic forces that reduce the coronary supply–demand ratio.

In that same publication [16], the following changes to the UDMI definition of Type 2 MI were proposed to reduce diagnostic variability:

(1) To align with the pathological data and the Braunwald unstable angina definition, epicardial causes of Type 2 MI should be included as a subcategory of Type 1 MI. Thus, Type 2 MI should be reserved for processes extrinsic to the coronary vascular bed.

(2) In patients presenting with a congestive heart failure (CHF) exacerbation and a small troponin increase, dyspnea as the only clinical evidence of myocardial ischemia should not automatically be considered indicative of Type 2 MI but rather of myocardial injury.

(3) In our estimation, the most fundamental problem with the Type 2 MI diagnosis is how to interpret a small increase in troponin above the upper reference limit and what constitutes a significant supply–demand mismatch. Both parameters are subjective and prone to personal interpretation. We hope that a fifth UDMI will address these issues to improve the standardization of Type 2 MI diagnosis.

### 3.4 Angiographic Findings in Type 2 NSTEMI

The diagnosis of Type 2 MI should be based on specific clinical criteria rather than coronary anatomy alone. Angiographic data in Type 2 MI indicate that significant coronary artery disease may be present in approximately 50–60% of cases, particularly in older patients with multiple comorbidities [14]. However, acute thrombotic culprit lesions caused by plaque rupture/erosion are not present [13]. In the remaining cases, in which coronary anatomy is evaluated, the findings may include non-obstructive disease or no identifiable plaque on invasive angiography. However, these estimates are imprecise, as most patients with Type 2 MI do not undergo coronary evaluation. This variable coronary anatomy likely contributes to the vagueness of current diagnostic criteria for Type 2 MI.

### 3.5 Angiographic Findings in Type 1 NSTEMI

In 1985, a prior report first described the angiographic characteristics of culprit lesions in unstable angina [19]. This work predated intravascular imaging, and little information was then known regarding the pathophysiology of unstable angina. We described that, in more than two-thirds of patients, the culprit lesion had severe obstruction and was eccentric on orthogonal views, with irregular borders, overhanging edges, or filling defects. These lesions were initially termed “type 2 eccentric” and later classified as complex lesions. Compared with culprit lesions in patients with stable angina, this complex lesion morphology was 71% sensitive and 84% specific for unstable angina. Based on a study by Levin and Fallon [20], irregular stenoses on post-mortem angiography in patients who died from MI had “complicated” histology characterized by plaque disruption and/or thrombus. Thus, it was postulated that these complex lesions identified *in vivo* on invasive angiography in unstable angina represented plaque disruption and/or intracoronary thrombus formation (Fig. 1, Ref. [19]).

**Fig. 1.**
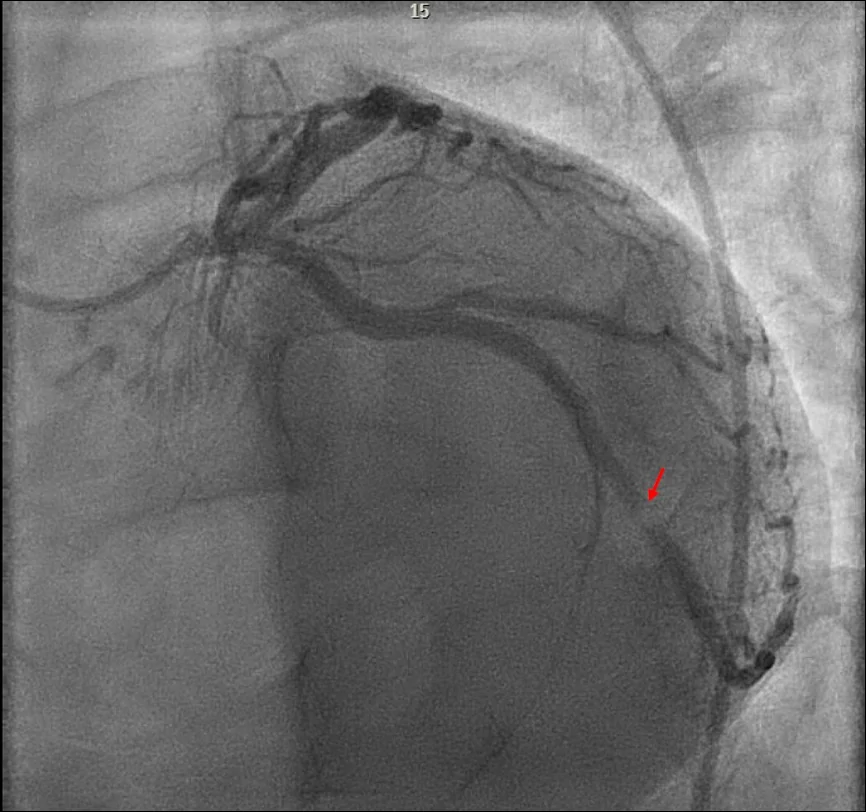
**Coronary angiogram from a patient who presented with a Type 1 NSTEMI**. The arrow highlights an eccentric culprit lesion with overhanging plaque edges in the distal left circumflex coronary artery (LCX). First described by Ambrose et al. (1985) [19], these lesions were classified as Type II eccentric lesions and were thought to represent plaque disruption, a partially occlusive thrombus, or both.

Clinically, unstable angina presented similarly to NSTEMI, but without elevation of cardiac enzymes consistent with AMI. With the introduction of troponin, a more sensitive marker than creatine kinase–myocardial band (CK-MB) for detecting myocardial necrosis in the 1980s, many patients previously described as having unstable angina would now be diagnosed with NSTEMI. As troponin assays became increasingly sensitive, the proportion of patients labeled with unstable angina declined further, prompting Braunwald and Morrow to publish a “requiem” for unstable angina in Circulation in 2013, noting its marginalization as ultrasensitive troponin assays converted most unstable angina presentations to NSTEMI [21].

What, then, are the angiographic findings in Type 1 NSTEMI? The answer depends on several factors, including age, sex, the number of risk factors, and the type of NSTEMI. A previous study assessed the angiographic findings in 193 patients with either Type 1 STEMI (n = 73) or NSTEMI (n = 120) [22]. In Type 1 NSTEMI, an identifiable culprit lesion was found in 56% of patients, and the majority (73%) of these lesions were complex, as described above. Non-obstructive/normal coronary arteries or 3-vessel disease were observed in 10% and 27% of patients, respectively. A culprit lesion could still be identified in 43% of those with 3-vessel disease.

At least one additional study attempted to identify the culprit lesion in patients with NSTEMI and multivessel disease using cardiac magnetic resonance imaging (MRI). In a prospective three-center study, 114 patients with first-presentation NSTEMI underwent delayed-enhancement cardiac MRI (DE-CMR) followed by coronary angiography [23]. With the interventionalist blinded to CMR findings, the infarct-related artery (IRA) was identifiable by angiography in 63% of cases. DE-CMR identified an IRA in 60% and also established a new non-CAD diagnosis in 19% of patients.

In our opinion, there are at least two NSTEMI presentations that are not classical but relatively common and must always be considered in any comprehensive discussion of NSTEMI.

#### 3.5.1 MINOCA

MINOCA accounts for roughly 5–10% of MI presentations and is an umbrella term encompassing several pathophysiologies, including atherosclerotic plaque disruption/erosion, epicardial or microvascular spasm, SCAD, and thromboembolism, after excluding non-ischemic mimics such as Takotsubo syndrome [24]. Invasive angiography and echocardiography are often inconclusive for establishing the underlying mechanism [8]. The PROMISE trial, a small investigation, evaluated a structured diagnostic strategy for patients with MINOCA. This approach included cardiac magnetic resonance (CMR) for tissue characterization, OCT when plaque instability was suspected, acetylcholine testing for vasomotor disorders, and echocardiography to search for embolic sources, followed by etiology-tailored therapy vs. standard care [24]. In this study, stratified management improved Seattle Angina Questionnaire summary scores by about 9 points at 12 months (a clinically important change) and identified a mechanism in 80% of patients. Epicardial spasm was most frequent (~36%), followed by plaque instability (~22%), SCAD (~13%), and embolism/microvascular spasm (each ~4%). The initial bedside impression could be reclassified in approximately 75% of cases. Although the study was underpowered for hard outcomes, major adverse cardiovascular events (MACEs) were numerically lower with stratified care, without added procedural harm, supporting the concept that mechanism-guided therapy is important in MINOCA. In practice, MINOCA should be considered only after non-ischemic causes (*e*.*g*., Takotsubo syndrome) have been excluded. However, we believe that MINOCA is sometimes overdiagnosed in clinical practice. Anecdotally, at our institution, several cases labeled as MINOCA were in fact compatible with Type 2 MI (unpublished observations). Whenever feasible, in the absence of an obvious Type 2 MI or another clear etiology, we believe additional testing—if available—should be pursued and may include CMR and/or intravascular or functional testing [24].

#### 3.5.2 SCAD

SCAD is a major non-atherosclerotic cause of ACS (often presenting as NSTEMI), especially in younger women and in those with peripartum status, fibromuscular dysplasia (FMD), or emotional/physical stress precipitants (Table 2) [25]. The underlying pathology is an intramural hematoma and/or an intimal tear compressing the true lumen. Clinically, SCAD more often presents as NSTEMI than STEMI. Large registries report roughly two-thirds NSTEMI and one-third STEMI (*e*.*g*., SCAD: ~70% NSTEMI and ~30% STEMI), with similar distributions in multicenter cohorts (*e*.*g*., 56% NSTEMI and 42% STEMI in a 389-patient series) [26]. Pregnancy-associated SCAD tends to present more frequently as STEMI [26].

**Table 2.** **Spontaneous coronary artery dissection (SCAD): angiographic types (Saw classification) and typical features [25]**.

SCAD type	Core angiographic appearance (what you see)	Key practical notes (why it matters)
Type 1 *Classic SCAD*	Multiple radiolucent lumens and/or contrast staining of the arterial wall with a visible dissection flap (“double lumen” appearance).	Most straightforward to recognize as SCAD on angiography. Can still be underappreciated if subtle or in small distal vessels.
Type 2 *Intramural hematoma pattern*	Long, diffuse, smooth narrowing (often >20–30 mm) with abrupt change in vessel caliber; may have distal tapering and can extend to the vessel tip (“2B” pattern often used when it reaches the distal segment).	Most common pattern. Frequently missed or misread as spasm. Intracoronary nitroglycerin may help exclude spasm (spasm improves; SCAD typically does not).
Type 3 *Focal/tubular stenosis*	Short, focal stenosis (often <20 mm) that mimics atherosclerotic plaque; lacks the classic long diffuse narrowing of Type 2.	Highest risk of misdiagnosis as atherosclerosis. Often requires intracoronary imaging (OCT/IVUS) for confirmation when safe and clinically necessary.
Type 4 *Complete occlusion*	Total occlusion of the artery (often distal) without an obvious atherosclerotic culprit; may appear as an abrupt cutoff.	Can resemble thrombotic occlusion/embolus. Distal occlusions are common; management is often conservative if flow can be restored or territory is small and patient is stable.

OCT, optical coherence tomography; IVUS, intravascular ultrasound.

In patients whose initial presentation mimics ACS but is ultimately attributed to SCAD, intravenous systemic anticoagulation is not routinely continued. While many patients receive unfractionated heparin (or other anticoagulants) early in the emergency department under standard ACS protocols, expert consensus recommends discontinuing anticoagulation once SCAD is suspected/confirmed [27]. Since SCAD is driven by an intramural hematoma and/or dissection plane, anticoagulation could theoretically promote hematoma expansion and propagation of the dissection. Therefore, anticoagulation should be reserved for clear alternative indications, such as atrial fibrillation with cardioembolic risk, left ventricular thrombus, venous thromboembolism, or mechanical valve, rather than used reflexively as part of routine ACS therapy in SCAD. In those rare situations, the risk–benefit balance may favor continued short-term anticoagulation with close monitoring. However, such decisions should be individualized and frequently reassessed in light of the competing risk of hematoma progression.

Coronary intervention in SCAD is technically challenging (because of the risk of wiring the false lumen and propagating the dissection). Invasive therapies are generally reserved for ongoing ischemia, left main involvement, or hemodynamic compromise. OCT or intravascular ultrasound (IVUS) should not be performed routinely in all suspected cases of SCAD; however, it should be considered when the diagnosis remains uncertain and the expected benefit outweighs the procedural risk. Many patients stabilize with conservative management alone, and spontaneous angiographic healing is common over weeks. Stenting is usually avoided unless there is ongoing ischemia and impaired blood flow distal to the SCAD that cannot be restored with balloon angioplasty alone. However, occasionally, stenting or even CABG may be required to salvage a large territory of myocardium, although overall mortality appears to be higher when CABG is performed.

Beta-blockers are used empirically to reduce shear stress; non-dihydropyridine calcium-channel blockers may be helpful for vasospastic symptoms. Statins are not routinely used unless dyslipidemia is present [27]. Patients should be screened for associated arteriopathies (*e*.*g*., iliac/renal/carotid fibromuscular dysplasia) and counseled on trigger avoidance and pregnancy planning. Recurrent events and residual symptoms are not uncommon and warrant the need for close follow-up and referral to cardiac rehabilitation [27,28].

## 4. Treatment of NSTEMI: Type 1 vs. Type 2

Since patients frequently present before the underlying mechanism is clear, distinguishing Type 1 (atherothrombotic plaque rupture/erosion) from Type 2 (oxygen supply–demand mismatch) NSTEMI is essential but can be clinically challenging during the initial evaluation. Some patients may be managed initially along standard ACS pathways until additional data (clinical context and/or routine blood tests; ECG/echo findings; troponin kinetics; if performed, coronary imaging) clarify the etiology [29]. However, when an obvious supply–demand mismatch is present, such as active bleeding, severe tachycardia, or hypotension due to sepsis, then the event should initially be managed as a Type 2 MI.

In Type 1 NSTEMI, treatment generally follows evidence-based ACS care: intravenous anticoagulation, antiplatelet therapy, and high-intensity statin therapy with additional guideline-directed anti-ischemic therapies as appropriate. An in-hospital invasive strategy is usually appropriate with coronary angiography and revascularization (PCI vs. CABG) guided by coronary anatomy, ischemic risk, comorbidity burden, and Heart Team considerations [29]. These are all Class 1 recommendations in the current guideline [29].

In contrast, management of Type 2 NSTEMI primarily involves treating the precipitating condition (*e*.*g*., sepsis, hypoxemia, tachyarrhythmia, hypertensive crisis, profound anemia, or active bleeding), with individualized decisions regarding antithrombotic therapy given the absence of a primary atherothrombotic event [30]. Importantly, many patients labeled as having Type 2 MI/NSTEMI have underlying CAD. Thus, decisions about whether to pursue invasive or coronary computed tomography angiography (CCTA), or only a noninvasive evaluation, should be individualized and consider several factors, such as the pre-test probability of obstructive CAD, symptom burden, hemodynamic stability, recurrent ischemia, ECG/imaging evidence of ischemia, bleeding risk, and overall clinical trajectory of each patient [30]. Based on the 2025 ACC/AHA ACS guidelines, when uncertainty exists, it is recommended to treat the event as Type 1 [29].

According to the guidelines, Class 1 recommendations for NSTE-ACS patients with Type 1 MI include parenteral anticoagulation for the index hospitalization. If unfractionated heparin is chosen, it should be continued for at least 48 hours or until PCI is performed with activated partial thromboplastin time (aPTT) monitoring. Fondaparinux reduces bleeding compared with enoxaparin in conservative strategies and is preferred when early PCI is not planned. If PCI is performed in a patient receiving fondaparinux, a single unfractionated heparin (UFH) bolus should be given to prevent catheter thrombosis. Enoxaparin is an acceptable alternative. Bivalirudin is reasonable in the cath lab, particularly if bleeding risk is high or if avoidance of GP IIb/IIIa inhibitors is desired [29].

### 4.1 DAPT in NSTEMI

In NSTE-ACS after PCI, aspirin plus either ticagrelor or prasugrel is generally preferred over clopidogrel based on superior ischemic outcomes and more rapid, potent platelet inhibition. Clopidogrel remains reasonable for lower bleeding risk/tolerance issues, in patients administering direct oral anticoagulants (DOACs), in the setting of drug–drug interactions, or cost/access limitations, and when CABG is likely [29].

Regarding early dual antiplatelet therapy (DAPT) before angiography, the 2023 European Society of Cardiology (ESC) ACS guideline is more restrictive, assigning a Class 3 (not recommended) indication against P2Y12 pretreatment when coronary anatomy is unknown and an early (<24 h) invasive strategy is planned. In contrast, the 2025 ACC/AHA ACS guideline is more permissive in a narrow scenario, allowing a weak Class 2b option to consider upstream clopidogrel or ticagrelor when an invasive strategy is planned; however, angiography is expected to be delayed (>24 h) [29,31]. If an ischemia-guided strategy is not elected or invasive angiography is delayed, pretreatment may be reasonable in selected, low-bleeding-risk patients with either clopidogrel or ticagrelor [29,30].

In high bleeding risk (HBR) patients, who comprise up to 40–45% of those undergoing PCI, individualized balancing of ischemic and hemorrhagic risk is essential. HBR is defined by the Academic Research Consortium (ARC-HBR) criteria as the presence of at least one major or two minor risk factors (*e*.*g*., oral anticoagulant use, severe chronic kidney disease (CKD), recent bleeding, advanced age, thrombocytopenia), corresponding to a ≥4% annual risk of Bleeding Academic Research Consortium (BARC) 3 or 5 bleeding [32]. Fig. 2 (Ref. [33]) lists high-bleeding and high-thrombotic-risk features [33]. High thrombotic-risk features are associated with higher ischemic risk. Clinicians must balance ischemic and bleeding risk in patients with overlapping risk features. Registry data confirm that patients with both high bleeding and high thrombotic risk experience the highest absolute rates of both MACEs and major bleeding, and neither current guidelines nor existing trials provide a clear, evidence-based DAPT strategy for this overlapping population. Individualized decision-making, ideally with serial reassessment of evolving risk, remains the most prudent approach [34].

**Fig. 2.**
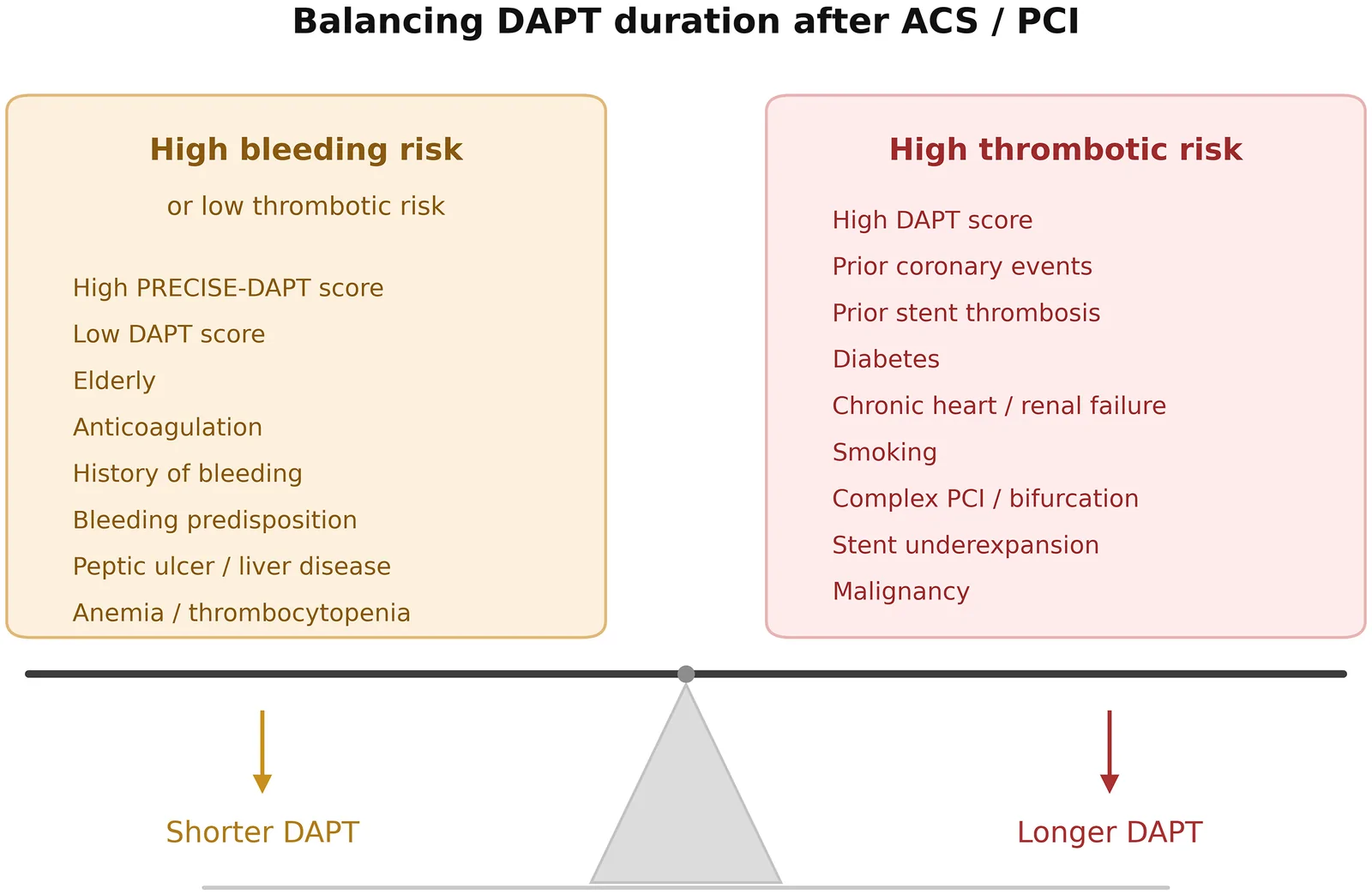
**Balancing DAPT duration [33]**. DAPT, dual antiplatelet therapy; ACS, acute coronary syndrome; PCI, percutaneous coronary intervention.

Both the 2025 ACC/AHA ACS guideline and the 2023 ESC ACS guideline recommend 12 months of DAPT as the default strategy for patients not at high bleeding risk (Class 1). For HBR patients, the two guidelines diverge modestly. The 2025 ACC/AHA ACS guideline provides a Class 1 recommendation for transitioning to ticagrelor monotherapy after ≥1 month of well-tolerated DAPT with ticagrelor post-PCI, supported by the TWILIGHT, TICO, and ULTIMATE-DAPT trials [29]. In contrast, the 2023 ESC guideline takes a more conservative approach, recommending P2Y12 inhibitor monotherapy after 3–6 months of event-free DAPT in HBR patients who are not at high ischemic risk (Class 2a, A). Abbreviation of DAPT to 1 month is denoted only as a Class 2b recommendation. Notably, both guidelines advise against reducing DAPT within the first 30 days after ACS. A recent meta-analysis of randomized trials in HBR patients further supports a shortened DAPT duration (1–3 months) followed by P2Y12 inhibitor monotherapy, with reduced bleeding without increased MACEs or stent thrombosis [35]. These considerations are particularly relevant when planning staged multivessel PCI, where cumulative contrast load, prolonged antiplatelet exposure, and procedural complexity compound bleeding risk. Another strategy of converting from ticagrelor to clopidogrel after one month (de-escalation) has also been studied [36]. In the 2025 ACC/AHA guidelines, de-escalation of DAPT after 1 month carries a weak Class 2b recommendation.

### 4.2 Invasive Strategy: When to Perform (and Defer) Angiography in Type 1 NSTEMI

According to the 2025 ACS guidelines, the timing of angiography in Type 1 NSTEMI should be guided by whether the patient meets very high-risk, high-risk, or intermediate-risk features (Class 1 or 2a recommendation). These criteria are summarized in Fig. 3 [29]. The timing of DAPT relative to angiography is discussed in the preceding section. Before elective CABG, an appropriate P2Y12 inhibitor washout period is required (clopidogrel/ticagrelor 3–5 days; prasugrel 7 days).

**Fig. 3.**
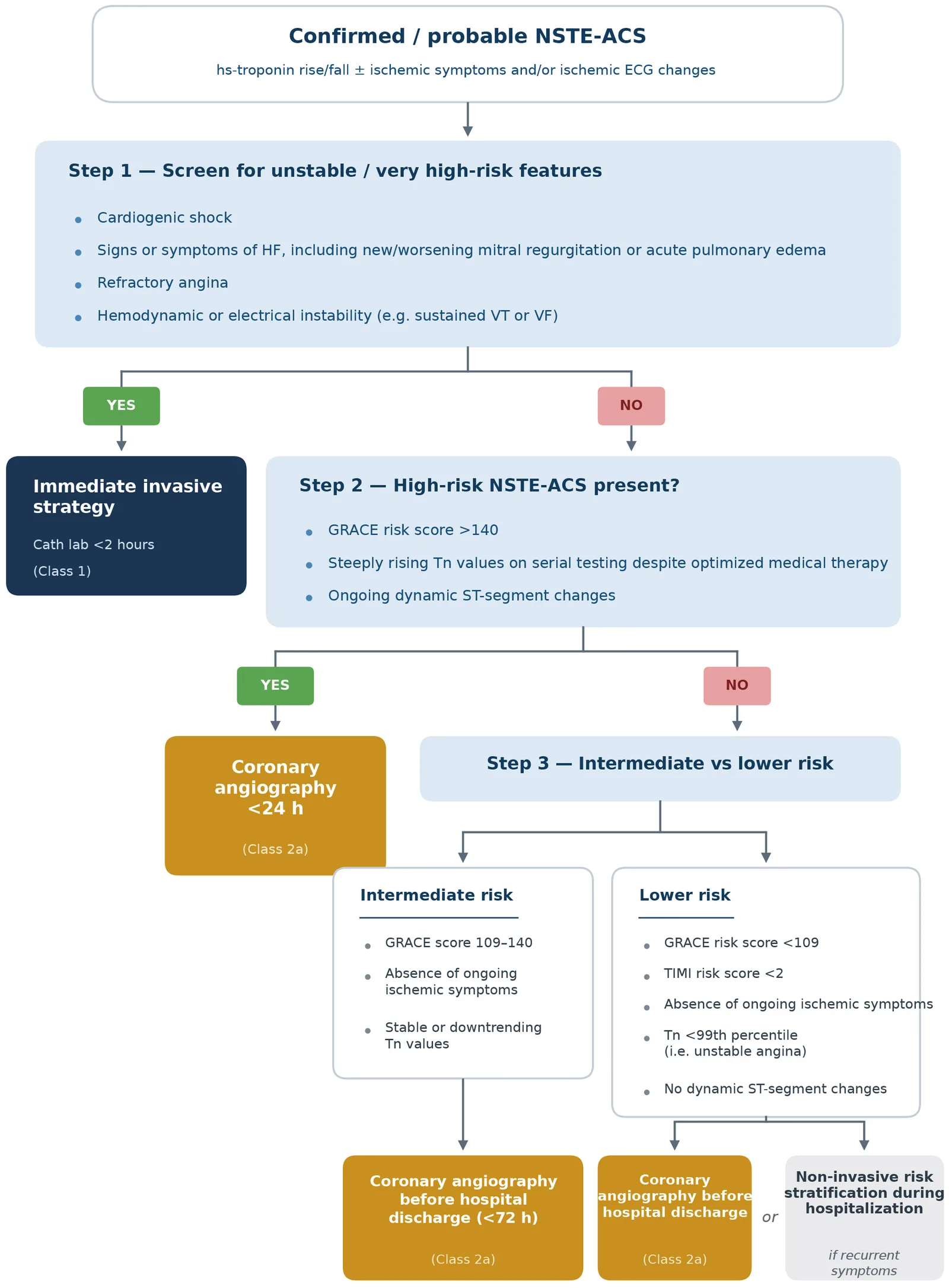
**Risk-stratified NSTEMI pathway for guiding the timing of invasive coronary angiography**. NSTE, non-ST-elevation; ACS, acute coronary syndrome; ECG, electrocardiogram; HF, heart failure; VT, ventricular tachycardia; VF, ventricular fibrillation; GRACE, Global Registry of Acute Coronary Events; TIMI, Thrombolysis in Myocardial Infarction.

### 4.3 PCI vs. CABG in NSTEMI

In NSTEMI with multivessel CAD, both the 2025 ACC/AHA ACS and the 2023 ESC ACS guidelines recommend complete revascularization. The choice between CABG and multivessel PCI should be guided by coronary complexity and comorbidities (ACC/AHA Class 1, ESC Class 2a) (Fig. 4) [29,37].

**Fig. 4.**
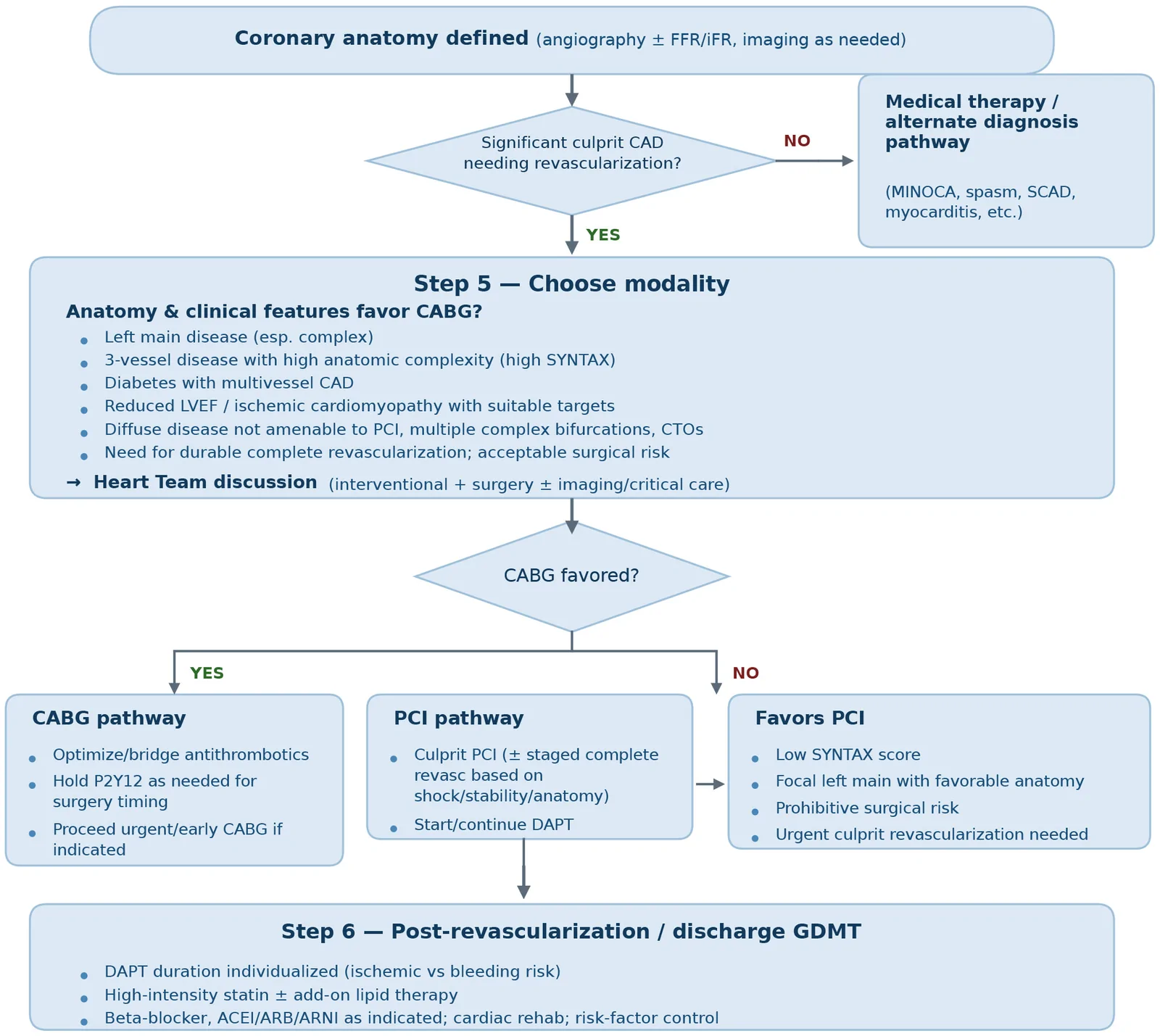
**PCI vs. CABG pathway**. CABG, coronary artery bypass grafting; GDMT, guideline-directed medical therapy. ACEI, angiotensin-converting enzyme inhibitor; ARB, angiotensin receptor blocker; ARNI, angiotensin receptor–neprilysin inhibitor; FFR, fractional flow reserve; iFR, instantaneous wave-free ratio; CAD, coronary artery disease; SCAD, spontaneous coronary artery dissection; MINOCA, myocardial infarction with non-obstructive coronary arteries; LVEF, left ventricular ejection fraction; CTO, chronic total occlusion; SYNTAX, Synergy between Percutaneous Coronary Intervention with TAXUS and Cardiac Surgery.

For left main disease, randomized trials demonstrate broadly comparable mid-term outcomes between CABG and PCI. However, PCI is associated with higher rates of repeat revascularization and, in some analyses, higher late death/MI, especially in people with diabetes with high SYNTAX scores [38,39,40]. Both guidelines support the physiological assessment of non-culprit stenoses to guide revascularization (ACC/AHA Class 2b; ESC Class 2b) and mandate a multidisciplinary Heart Team approach that integrates coronary anatomy, left ventricular function, diabetes, frailty, stroke risk, and patient preference. In older or frail patients, viability testing, overall comorbidity burden, and goals of care often limit surgical plans. In such cases, culprit-only PCI, staged PCI, or hybrid strategies (culprit PCI initially, followed by CABG) remain reasonable options when clinical stability permits [39,40].

### 4.4 Complete vs. Culprit-Only Revascularization in NSTEMI With PCI

For STEMI, several large randomized controlled trials (RCTs) (PRAMI, DANAMI-3-PRIMULTI, COMPLETE) support complete revascularization of suitable non-culprit lesions, usually in a staged fashion, thereby reducing reinfarction and repeat revascularization [40,41,42]. However, in Type 1 NSTEMI, there is no large, dedicated randomized clinical trial definitively establishing a complete-versus-culprit PCI strategy. While CABG typically achieves complete revascularization, evidence for complete revascularization with PCI is based on smaller trials and observational/meta-analytic data. The SMILE trial, a small, two-center study, suggested that complete revascularization (often staged) reduces the need for repeat ischemia/revascularization without clear safety penalties. In the SMILE trial (584 patients, two-center, unblinded), single-stage complete revascularization reduced the 1-year composite major adverse cardiac and cerebrovascular events (MACCE) endpoint by approximately 45% compared with multistage PCI (13.6% vs. 23.2%), driven primarily by a 48% relative reduction in target vessel revascularization (8.3% vs. 15.2%). No significant differences were observed in the individual hard endpoints of death or reinfarction [43].

Additional data supporting complete revascularization were identified from FAMOUS-NSTEMI, a physiology-guided NSTEMI strategy, which showed that fractional flow reserve (FFR) can safely defer non-ischemic lesions and reduce stent use without worsening outcomes, implying that “functionally complete” rather than purely angiographically complete revascularization may be optimal [44]. Contemporary meta-analyses and registries generally favor treating significant non-culprit disease, either during the index procedure or staged, when lesion severity is supported by physiology or clear ischemia, with approximately 27–47% relative reductions in composite MACEs compared with culprit-only PCI [45]. Accordingly, the 2025 ACS guidelines provide a Class 1 recommendation in stable patients with NSTE-ACS and multivessel disease (MVD), without left main stenosis, who are not intended for CABG and who undergo culprit-lesion PCI: PCI of significant non-culprit lesions (at the time of the index procedure or as a staged procedure) is recommended to reduce the risk of MACEs [29].

**Who benefits most from complete PCI?** Younger patients with few comorbidities, clear multivessel ischemia, and complex culprit biology (*e*.*g*., large plaque burden) tend to benefit from a planned, staged, complete approach. **Who might not?** Older, frail patients or those with severe CKD, anemia, high bleeding risk, or tortuous/diffuse small-vessel disease may be better served with culprit-only PCI plus optimal medical therapy, reserving non-culprit revascularization for refractory symptoms or objective ischemia (opinion-based). In practice, a Heart Team-based, physiology-guided, staged strategy is most consistent with current guidance while we await a definitive NSTEMI-specific RCT [29,30,31,37,38,39,40,41].

When PCI is pursued in the NSTEMI setting, particularly for complex or multivessel disease, contemporary evidence increasingly supports the use of intravascular imaging (IVUS or OCT) to guide procedural decision-making [46]. This is a Class 1 recommendation in the 2025 ACS guidelines. Intravascular imaging can optimize stent sizing, characterize plaque morphology and composition, confirm adequate stent expansion and apposition, and detect complications such as edge dissection or tissue protrusion that may not be apparent on angiography alone. These modalities are particularly valuable in NSTEMI, where culprit lesion identification may be challenging, and in cases of suspected SCAD, when OCT is used selectively to distinguish dissection flaps from atherosclerotic disease [46].

## 5. Conclusion

NSTEMI is a heterogeneous clinical syndrome that encompasses a spectrum of mechanisms, including atherothrombotic plaque disruption (rupture, erosion, and calcified nodules), dynamic epicardial or microvascular obstruction, spontaneous coronary artery dissection, thromboembolism, and supply–demand imbalance; each mechanism has distinct diagnostic pathways and therapeutic implications. Moving from a broad syndromic label to a personalized mechanism-specific diagnosis is imperative. In selected cases, standard evaluation may be insufficient, and more advanced diagnostic tools, if available, may be needed, as described in patients with MINOCA. However, in the vast majority of cases, good clinical judgment, along with standard diagnostic and therapeutic tools, should be sufficient to establish an appropriate diagnosis and guide effective treatment.

## 6. Future Developments

For atherosclerotic disease and Type 1 MI, prevention is essential. Earlier detection and treatment of atherosclerosis are critical to significantly reduce Type 1 MI (both STEMI and NSTEMI). Clinicians should aim to identify at-risk patients earlier in the disease course through appropriate screening and risk-factor modification. Potential approaches include coronary artery calcium scoring and, ideally, CT angiography or other techniques in appropriately selected high-risk patients. CT angiography can identify asymptomatic plaque burden and plaque characteristics, particularly low-attenuation plaque. In the multicenter SCOT-HEART trial, low-attenuation noncalcified plaque burden on CCTA emerged as the strongest independent predictor of future fatal or nonfatal myocardial infarction, outperforming cardiovascular risk scores, coronary artery calcium scoring, and stenosis severity. Patients with low-attenuation plaque burden exceeding 4% had an almost 5-fold increased risk of subsequent MI [47,48,49]. A recent expert consensus statement from the Quantitative Cardiovascular Imaging Study Group provided evidence-based recommendations for integrating CCTA with artificial intelligence (AI)-supported plaque analysis to individualize treatment based on atherosclerotic plaque characterization, highlighting low-attenuation plaque and positive remodeling as key features for cardiovascular risk assessment [50].

Lifelong attention to modifiable risk factors, including hypertension, diet/exercise/obesity, low-density lipoprotein levels, avoidance of smoking/other pollutants, and glucose control (HbA1c) in patients with diabetes, is essential and should be a priority for anyone who values optimal cardiovascular health. Patients with a family history of premature myocardial infarction, stroke, or sudden death in adulthood should also have lipoprotein(a) measured. Recent guidelines recommend screening for atherosclerosis beginning at age 30 years, using the PREVENT Risk Calculator (refer clinicians to contemporary lipid and hypertension guidelines for additional recommendations) [51].

### 6.1 Artificial Intelligence in Diagnosis and Management

AI holds considerable promise for earlier detection, more precise risk stratification, and improved imaging interpretation; however, AI also requires prospective validation, equitable performance across populations, and smooth integration into clinical workflows [52,53,54,55]. Potential applications include automated interpretation of symptoms and ECGs, serial biomarker testing to enable earlier diagnosis or safe early discharge, and AI-based plaque characterization using intravascular or noninvasive imaging [56,57]. Barriers to widespread clinical adoption include the need for robust external validation across diverse populations, integration into existing care pathways, clinician interpretability, and the risk of bias. Current guidelines acknowledge the promise of AI but stop short of recommending routine adoption. Ongoing prospective studies will determine whether AI improves actionable outcomes, including time to therapy, bleeding-adjusted net clinical benefit, and resource utilization [51].

### 6.2 Unanswered Questions

#### 6.2.1 Plaque Erosion: A Distinct Mechanism Requiring Tailored Management

Unlike plaque rupture, which involves disruption of a thin fibrous cap overlying a macrophage-infiltrated, lipid-rich necrotic core, plaque erosion is characterized by endothelial denudation over a proteoglycan- and smooth muscle cell-rich plaque without cap disruption. Moreover, plaque erosion is driven by innate immune mechanisms, including Toll-like receptor 2 (TLR2) activation, neutrophil extracellular trap (NET) formation, and hyaluronan degradation, and results in a predominantly platelet-rich (white), nonocclusive thrombus [58,59]. Erosion accounts for approximately 25–40% of ACS cases in younger patients, women, and smokers with fewer traditional risk factors, and represents a majority of coronary thrombi in women under 50 years of age [60]. Currently, in addition to a healthy lifestyle, there is no consensus on strategies to prevent plaque erosion. Further studies are required.

#### 6.2.2 Invasive Strategy in Type 2 NSTEMI

Type 2 NSTEMI is associated with higher in-hospital and post-discharge mortality than Type 1 NSTEMI [14]. While patients with Type 2 NSTEMI have more comorbidities, current data do not establish CAD as the cause of the higher mortality. However, CAD is often present among patients undergoing invasive evaluation. These patients are routinely excluded from ACS intervention trials, leaving no randomized evidence to guide whether, when, or in whom angiography and/or revascularization should be performed. Observational data suggest that a subset of patients with underlying obstructive CAD may benefit from invasive evaluation once the inciting trigger is corrected, but reliably identifying this subgroup remains challenging [30]. Prospective trials are needed to determine which Type 2 NSTEMI patients warrant coronary angiography or revascularization, and to define the optimal timing relative to the precipitating condition.

#### 6.2.3 Prasugrel Versus Ticagrelor

Both prasugrel and ticagrelor are preferred over clopidogrel in ACS patients undergoing PCI, yet guidelines diverge in their recommendations. The 2023 ESC guidelines assign prasugrel a Class 2a preference based primarily on the open-label ISAR-REACT 5 trial, whereas the 2025 ACC/AHA guidelines consider prasugrel and ticagrelor interchangeable (Class 1), citing design limitations that constrain the generalizability of a single study [29,31,61,62,63]. Prasugrel remains contraindicated in patients with prior stroke/transient ischemic attack (TIA) and requires dose reduction in older or low-weight patients. In contrast, ticagrelor is the only potent P2Y12 inhibitor with Class 1 guideline support for medically managed NSTE-ACS without planned PCI [29,63]. A definitive double-anonymized head-to-head trial is still needed.

#### 6.2.4 DAPT After Drug-Coated Balloon (DCB) Angioplasty

DCBs deliver drugs without leaving a permanent scaffold. Trials such as BASKET-SMALL 2 and DEBUT support their efficacy in small-vessel disease and in patients at HBR [64,65,66,67]. One randomized trial demonstrated the non-inferiority of stepwise DAPT de-escalation compared with standard 12-month DAPT in ACS patients treated exclusively with DCB [68]. The International DCB Consensus Group endorses only 4 weeks of DAPT after DCB-only angioplasty. Nevertheless, current ESC guidelines still extrapolate longer DAPT durations from stent-based data, underscoring the need for larger confirmatory trials [31,64].

#### 6.2.5 Updated Nomenclature for STEMI/NSTEMI?

Over the years, the nomenclature for what one now refers to as NSTEMI has evolved. As outlined in the historical perspective, the original terminology was non-transmural MI. It then changed over time to subendocardial or non-Q-wave MI and finally NSTEMI. New data suggests that further evolution should be considered. It has been shown that NSTEMI with total coronary occlusion of the infarct-related artery has a worse prognosis than NSTEMI without total occlusion but similar to STEMI with total occlusion [69]. Thus, nomenclature should now reflect the presence of total occlusion as it provides a more accurate classification of STEMI/NSTEMI. However, the authors of this review do not necessarily concur that new terminology is required as in a large percentage of NSTEMI, one is unable to assign an infarct-related artery.
